# Supplementary material for: Interacting with volatile environments stabilizes hidden-state inference and its brain signatures
Source: Nat Commun. 2021 Apr 13;12:2228. doi: 10.1038/s41467-021-22396-6 (PMC8044147; doi:10.1038/s41467-021-22396-6)
Supplement: Supplementary file 3 — Reporting Summary [file 41467_2021_22396_MOESM3_ESM.pdf]

nature research

Corresponding author(s): Valentin Wyart

Last updated by author(s): 2021/02/16

# Reporting Summary

Nature Research wishes to improve the reproducibility of the work that we publish. This form provides structure for consistency and transparency in reporting. For further information on Nature Research policies, see our Editorial Policies and the Editorial Policy Checklist.

## Statistics

For all statistical analyses, confirm that the following items are present in the figure legend, table legend, main text, or Methods section.

| n/a | Confirmed | |
|---|---|---|
| ☐ | ☒ | The exact sample size (*n*) for each experimental group/condition, given as a discrete number and unit of measurement |
| ☐ | ☒ | A statement on whether measurements were taken from distinct samples or whether the same sample was measured repeatedly |
| ☐ | ☒ | The statistical test(s) used AND whether they are one- or two-sided<br>*Only common tests should be described solely by name; describe more complex techniques in the Methods section.* |
| ☐ | ☒ | A description of all covariates tested |
| ☐ | ☒ | A description of any assumptions or corrections, such as tests of normality and adjustment for multiple comparisons |
| ☐ | ☒ | A full description of the statistical parameters including central tendency (e.g. means) or other basic estimates (e.g. regression coefficient) AND variation (e.g. standard deviation) or associated estimates of uncertainty (e.g. confidence intervals) |
| ☐ | ☒ | For null hypothesis testing, the test statistic (e.g. $F$, $t$, $r$) with confidence intervals, effect sizes, degrees of freedom and $P$ value noted<br>*Give P values as exact values whenever suitable.* |
| ☐ | ☒ | For Bayesian analysis, information on the choice of priors and Markov chain Monte Carlo settings |
| ☒ | ☐ | For hierarchical and complex designs, identification of the appropriate level for tests and full reporting of outcomes |
| ☐ | ☒ | Estimates of effect sizes (e.g. Cohen's $d$, Pearson's $r$), indicating how they were calculated |

*Our web collection on statistics for biologists contains articles on many of the points above.*

## Software and code

Policy information about availability of computer code

| | |
|---|---|
| Data collection | MEG data was recorded using a whole-head Elekta Neuromag TRIUX system (Elekta Instrument AB, Stockholm, Sweden). |
| Data analysis | The preprocessing pipeline was implemented using the FieldTrip toolbox (http://www.fieldtriptoolbox.org, version 2018/04/04), and additional custom scripts written in MATLAB (Mathworks). The particle MCMC model fitting procedure was written in Julia v1.0 (https://julialang.org). The random-effects Bayesian model selection procedure was implemented in SPM12 (Wellcome Center for Human Neuroimaging; http://www.fil.ion.ucl.ac.uk/spm). Cortical surface-based segmentation and reconstruction was performed with the FreeSurfer image analysis suite (http://surfer.nmr.mgh.harvard.edu, version 6). MEG source reconstruction was performed with Brainstorm (http://neuroimage.usc.edu/brainstorm, version 191115). The custom scripts used to analyze the behavioral and MEG data are available from the corresponding authors upon request. |

For manuscripts utilizing custom algorithms or software that are central to the research but not yet described in published literature, software must be made available to editors and reviewers. We strongly encourage code deposition in a community repository (e.g. GitHub). See the Nature Research guidelines for submitting code & software for further information.

## Data

Policy information about availability of data

All manuscripts must include a data availability statement. This statement should provide the following information, where applicable:

- Accession codes, unique identifiers, or web links for publicly available datasets
- A list of figures that have associated raw data
- A description of any restrictions on data availability

The manuscript includes all datasets generated or analyzed during this study. The behvioral data from the main magnetoencephalography experiment and the

control behavioral experiment are publicly available at https://doi.org/10.6084/m9.figshare.13200128. Source data are provided with this paper. The magnetoencephalography data are available from the corresponding authors upon request.

# Field-specific reporting

Please select the one below that is the best fit for your research. If you are not sure, read the appropriate sections before making your selection.

☐ Life sciences ☒ Behavioural & social sciences ☐ Ecological, evolutionary & environmental sciences

For a reference copy of the document with all sections, see nature.com/documents/nr-reporting-summary-flat.pdf

# Behavioural & social sciences study design

All studies must disclose on these points even when the disclosure is negative.

| | |
|---|---|
| Study description | Tested participants were asked to play a reversal learning task based on visual stimuli which we framed, in two conditions corresponding to different blocks of trials, either as cue-based or outcome-based inference. Their behavior and magnetic brain activity recorded in magnetoencephalography (MEG) was modeled using a Bayesian inference process and analyzed in a quantitative fashion to compare reversal learning behavior and brain activity between the cue-based and outcome-based conditions. |
| Research sample | 24 adult participants took part in the main magnetoencephalography study contrasting cue-based and outcome-based inference (12 females, mean age: 24 years, age range: 20–30 years, all right-handed). 30 additional adult participants took part in the control behavioral study contrasting retrospective and prospective cue-based inference (5 excluded due to chance-level performance in more than one block of trials, 15 females in the retained sample, mean age: 26 years). Participants had no history of neurological and psychiatric disease and had normal or corrected-to-normal vision. Given the absence of prior effect sizes for the difference between 'observation' (cue-based) and 'action' (outcome-based) conditions, we chose a sample size (n = 24) which exceeded the average sample size used in human MEG studies at the time of data collection. |
| Sampling strategy | The eight blocks of the main task were organized in pairs of blocks of the cue-based and outcome-based conditions, whose order was counter-balanced both within and between participants. The counter-balancing of the different aspects of the task across participants required to test a multiple of n = 8 participants. Given the absence of prior effect sizes for the difference between cue-based and outcome-based conditions, we chose a sample size (n = 24) which exceeded the average sample size used in human MEG studies at the time of data collection. All results presented in the main text collapse across the two types of blocks within each condition, unless noted otherwise. |
| Data collection | MEG data was recorded using a whole-head Elekta Neuromag TRIUX system (Elekta Instrument AB, Stockholm, Sweden) composed of 204 planar gradiometers and 102 magnetometers, at a sam-pling frequency of 1,000 Hz. Visual fixation was monitored online throughout the main task using an EyeLink 1000 eye-tracking system (SR Research, Ottawa, Canada), using a monocular tracking of the dominant eye at a sampling frequency of 1,000 Hz. Data collection was performed in the presence of the experimenters and the data collection technician of the MEG imaging center. Neither the experimenters nor the participants were blind to the experimental conditions being tested, which corresponded to explicit instructions. |
| Timing | The data for the MEG experiment (N = 24 participants) was collected between September and December 2015. The data for the control behavioral experiment (N = 30 participants) was collected between February and April 2019. |
| Data exclusions | No subject was excluded from the MEG experiment. N = 5 subjects were excluded from the control behavioral experiment due to chance-level performance in more than one block of trials. The same exclusion criterion has been applied to both experiments, and was pre-determined at the time of data collection for the behavioral experiment based on the analysis of the prior MEG experiment. |
| Non-participation | No participant dropped out or cancelled their participation to the experiments. |
| Randomization | We used a within-subject design, where the order of conditions being tested were counter-balanced and randomized across participants. The cue-based and outcome-based conditions were distinct in the instructions provided to the participants, and they were thus by definition not blind to participants. |

# Reporting for specific materials, systems and methods

We require information from authors about some types of materials, experimental systems and methods used in many studies. Here, indicate whether each material, system or method listed is relevant to your study. If you are not sure if a list item applies to your research, read the appropriate section before selecting a response.

## Materials & experimental systems

| n/a | Involved in the study |
| --- | --- |
| ☒ | ☐ Antibodies |
| ☒ | ☐ Eukaryotic cell lines |
| ☒ | ☐ Palaeontology and archaeology |
| ☒ | ☐ Animals and other organisms |
| ☐ | ☒ Human research participants |
| ☒ | ☐ Clinical data |
| ☒ | ☐ Dual use research of concern |

## Methods

| n/a | Involved in the study |
| --- | --- |
| ☒ | ☐ ChIP-seq |
| ☒ | ☐ Flow cytometry |
| ☒ | ☐ MRI-based neuroimaging |

# Human research participants

Policy information about studies involving human research participants

| | |
| --- | --- |
| Population characteristics | See above. |
| Recruitment | Participants were recruited through online posting on the public mailing list from the Relais d'Information en Sciences Cognitives (RISC) in France. Subscribers to the public mailing list are typically young adults (university students in particular) in the age range that was targeted for our experiment (20-30 years). |
| Ethics oversight | All tested participants gave a written informed consent before taking part in the study, which re-ceived ethical approval from relevant authorities (Comité de Protection des Personnes Ile-de-France VI, ID RCB: 2007-A01125-48, 2017-A01778-45). |

Note that full information on the approval of the study protocol must also be provided in the manuscript.
